# Different Dimensions of Social Support on Social Media and Leisure-Time Physical Activity Intentions Among Chinese College Students Applying the Theory of Planned Behavior: A Cross-Sectional Study

**DOI:** 10.2196/90020

**Published:** 2026-06-23

**Authors:** Ni Zhang, Carolyn Madison Chang, Yu Zhang, Lu Guo, Qizhi Xu

**Affiliations:** 1 Department of Public Health, San Jose State University, San Jose, CA, United States; 2 Department of Communication of Science and Technology, School of Humanities and Social Sciences, University of Science and Technology of China, 96# Jinzhai Road, Hefei, Anhui, 230026, China, 86 13155108598; 3 School of Literature and Arts, Southwest University of Science and Technology, Mianyang, Sichuan, China

**Keywords:** social support on social media, companionship support, esteem support, informational support, leisure-time physical activities, theory of planned behavior, TPB, Chinese college students

## Abstract

**Background:**

Leisure-time physical activity (LTPA) participation among Chinese college students remains insufficient despite growing public health concerns regarding sedentary lifestyles and obesity. Social media platforms have increasingly become important channels for delivering social support related to physical activity (PA); however, different dimensions of social support on social media may exert distinct psychological influences on exercise intentions.

**Objective:**

Guided by the theory of planned behavior, this study aimed to examine the direct and indirect relationships between different dimensions of social support on social media and Chinese college students’ intentions to engage in LTPA.

**Methods:**

A cross-sectional online survey was conducted among undergraduate and graduate students from a comprehensive university in East China between February and June 2022. A total of 310 valid responses were analyzed using structural equation modeling in R. Three dimensions of social support on social media, including companionship support, informational support, and self-esteem support, were examined as predictors of LTPA intention, with the theory of planned behavior constructs (attitude, subjective norms, and perceived behavioral control) modeled as mediators. Demographic characteristics, BMI, social media use, and prior PA behaviors were included as covariates.

**Results:**

Companionship support demonstrated the strongest positive association with LTPA intention, including both a direct effect (β=.348; *P*=.005) and an indirect effect through attitudes toward PA (β=.286; *P*=.002). Self-esteem support also showed a significant positive indirect association with LTPA intention via attitude (β=.138; *P*=.02). In contrast, informational support demonstrated a significant negative indirect effect on LTPA intention through attitude (β=−.291; *P*<.001). Subjective norms and perceived behavioral control did not significantly mediate the relationships between social support dimensions and LTPA intention. The findings suggest that companionship-oriented interactions on social media may strengthen positive exercise attitudes, whereas excessive or low-credibility informational content may undermine exercise motivation.

**Conclusions:**

Different dimensions of social support on social media play distinct roles in shaping Chinese college students’ intentions to engage in LTPA. Social media–based PA interventions should prioritize companionship and esteem support while improving the credibility, personalization, and quality of informational support to reduce potential negative effects associated with misinformation and information overload.

## Introduction

### Background

Leisure-time physical activity (LTPA) refers to voluntary recreational physical activities (PAs; eg, sports, physically active hobbies, exercise) [1]. Participation in these activities is linked to numerous favorable physical health outcomes, such as a reduced risk of all-cause mortality, healthier gut microbiomes, attenuated biological aging, and reduced allostatic load [2-6]. Findings from the 2023 College Health Tracking Survey indicate that only 30.2% of Chinese university students reported participating in moderate-to-vigorous PA within the past month, underscoring the low prevalence of PA participation [7]. Furthermore, a secular analysis conducted by Sun et al [8] revealed overall declines in standardized fitness scores between 2015 and 2023.

### Barriers to LTPA Participation Among College Students in China

Researchers have noted that secondary and higher educational systems in China are structured to reinforce cultural beliefs that academic achievement is paramount [9]. The range of educational and career pathways available to individual students is largely determined or even constrained by performance metrics such as entrance exam scores and course grades. For example, undergraduate admissions are contingent upon performance in a nationally administered exam known as “Gaokao,” an event that plays a central role in Chinese society for its perceived importance for social mobility and life trajectories [10]. Existing research has shown that the cultural premium placed on academic performance negatively affects health-related behaviors. Particularly amid post-COVID-19 employment uncertainty, Chinese university students frequently cite academic pressure and perceived time constraints as the principal barrier to PA engagement [11]. Resource limitations, such as limited access and inadequate professional guidance for PA, also contribute to low exercise engagement on Chinese university campuses. Although the number of publicly available sports venues per capita has been steadily increasing since 2000, researchers have noted that access is still insufficient to meet demand [11]. Moreover, the absence of widely available professional guidance around attaining specific exercise goals deters students from participation, potentially reflecting limited self-efficacy [11,12]. Social support may mitigate some of these resource limitations, as it has been found to bolster self-efficacy and is linked to PA participation among Chinese students [12,13].

### Social Support and Its Dimensions

Social support is highly valued in Chinese culture and has been consistently associated with PA behaviors. A cross-sectional study in Inner Mongolia in China found that a 1-unit increase in perceived social support on the Likert scale was associated with a 15% increase in the likelihood of participating in PA [13]. In the collectivist culture of China, individuals are expected and encouraged to depend on close social networks for support [14].

Social support is typically conceptualized as comprising 3 core dimensions: companionship support (engaging in activities together), informational support (providing advice or knowledge), and esteem support (offering encouragement and confidence) [15,16]. Each dimension of social support can play unique roles in promoting LTPA among college students. For instance, parents may offer instrumental assistance in the form of transportation or exercise equipment, while peers more often provide companionship by involving one another in collective LTPA [17]. As students transition to college, support from friends and classmates often becomes more strongly influential on PA intentions, whereas parental and family support diminishes in effect [17,18]. Within Chinese university settings, institutional support is sometimes available as access to sports facilities and supportive campus cultures, both of which have also been found to play a vital role in enhancing exercise engagement [19].

### Social Support on Social Media

Social media platforms, such as WeChat, Weibo, RedNote, and Douyin, are widely used by Chinese college students. Chinese social networking sites enable users to connect with friends and family, as well as strangers, businesses, and news outlets [20]. Social media platforms allow users to seek and offer both companionship support and informational support, thereby increasing the likelihood of PA participation [21].

However, the proliferation of information and user-generated content on social media can negatively affect PA participation. As any user can post anything on social media, LTPA-related misinformation may be misrepresented as valid informational support, which can diminish the motivation to exercise [22]. In addition, studies have found that prolonged exposure to high volumes of social media information can cause information overload, which can manifest as elevated social media fatigue, increased levels of overwhelm, heightened anxiety levels, and reduced self-efficacy [23-25].

The mechanisms through which different dimensions of social support on social media influence Chinese college students’ intentions to participate in PA remain understudied. Innovatively applying the theory of planned behavior (TPB), this study aims to fill this gap by exploring the sociopsychological mechanisms through which social support on social media shapes LTPA intentions among Chinese college students.

### Theory of Planned Behavior

#### Overview

The TPB is a theoretical framework that identifies 3 variables—attitude, perceived behavioral control, and subjective norms—as key determinants of individual behavioral intention [26]. TPB is often used to scaffold and guide research hypotheses about behavioral change and intervention effectiveness [27,28].

#### Attitude

Attitude reflects an individual’s appraisal of the value associated with performing a specific behavior and is positively associated with PA participation [26]. Research on the social features of mobile fitness apps finds that both attitude and perceived behavioral control are positively associated with exercise intentions among Chinese adults [29,30].

#### Perceived Behavioral Control

Perceived behavioral control (PBC) is an individual’s evaluation of their own ability, informed by cognitive appraisals of various perceived barriers and facilitators, to perform a particular behavior [26,28]. As a TPB construct, PBC tends to be most strongly associated with behavioral intention among Chinese college students regardless of age [31]. Self-efficacy (one’s perceived capability to perform a behavior) is a core component of perceived behavioral control in TPB [12,17].

#### Subjective Norms

Subjective norms describe the perceived social pressures surrounding a behavior [26]. Some researchers have found that subjective norms are often weakly associated with PA. Researchers have noted that subjective norms have a far more limited predictive relationship with PA compared to other TPB constructs [27,29].

### Relationships Between Various Dimensions of Social Support and LTPA Intention Based on TPB

Some studies have found indirect relationships between social support and PA via both attitude and PBC. For example, Zhang et al [28] found that social support for LTPA from contacts on social media has an indirect effect on intention through attitude and perceived PBC among college students in the United States. One study among Chinese college students observed that social support significantly predicted self-efficacy, which—in turn—strongly mediated the relationship between social support and PA, accounting for nearly 60% of the total effect [12].

Other studies have found indirect effects of different dimensions of social support via either attitude or PBC (or self-efficacy) alone. For example, informational support was found to have a strong indirect association with exercise intentions through the mediation of attitudes toward exercise among Chinese fitness-app users [30]. However, as any user can post anything on social media, low-quality or misleading information can be presented in ways that resemble legitimate informational support [22]. However, various studies confirm that social support fosters greater self-efficacy, thus increasing the likelihood of exercise adherence [12,17,32]. Wang and Zhao [33] observed that Chinese college students who scored lower on Exercise Self-Efficacy Scales benefitted the most from social support, thus suggesting that social support buffers against the potential negative impacts of low self-efficacy.

However, few studies have tested the direct and indirect links between various dimensions of social support on social media and LTPA intention among Chinese college students applying the TPB. We aim to fill this gap in our study. Specifically, our hypotheses are as follows:

- H1: (1) Companionship support, (2) informational support, and (3) self-esteem support are directly associated with Chinese college students’ LTPA intentions.
- H2: TPB variables, including (1) attitude, (2) subjective norms, and (3) PBC, mediate the relationships between (1) companionship support, (2) informational support, and (3) self-esteem support and exercise intentions.

**Figure 1.**
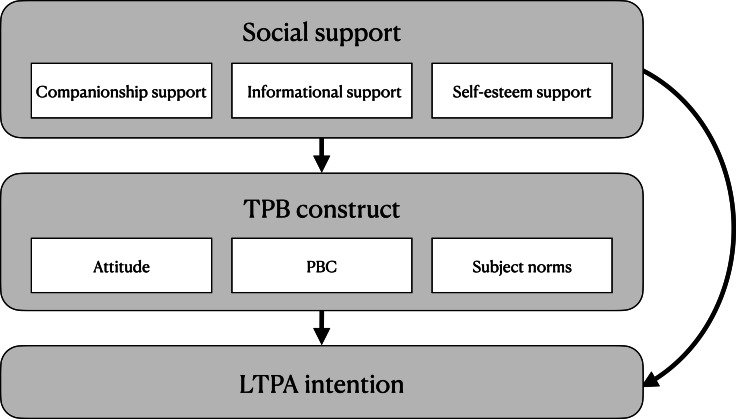
Theoretical model with the theory of planned behavior as mediator. LTPA: leisure-time physical activity; PBC: perceived behavioral control; TPB: theory of planned behavior.

The conceptual model is shown in Figure 1.

## Methods

### Data Collection

Participants were recruited from a comprehensive university in East China using convenience sampling. Eligible participants were undergraduate or graduate students currently enrolled at the university and aged 18 years or older. The survey was administered online via Qualtrics, and the survey link was distributed through WeChat. The study protocol (including both English and Chinese versions) was approved by the Institutional Review Board (IRB) of San Jose State University in the United States. Participants provided informed consent at the start of the survey before answering any questions.

The data were collected between February and June 2022. A total of 430 students accessed the survey, of whom 328 completed the questionnaire. The median questionnaire completion time was 556 (IQR 508-870) seconds. Participants with a completion time less than one-third of the median were excluded. Responses were screened for data quality by removing cases with substantial missing data (>20% of the items unanswered) or uniform responses across items. After data cleaning, 310 valid responses were retained for all analyses, yielding an effective response rate of 72.1%.

In the final valid sample, 76 participants were recruited through course announcements in classrooms, and 234 were recruited at the entrance of the university cafeteria by trained research assistants. To ensure consistent recruitment quality across the 2 settings, all research assistants received identical standardized training on eligibility criteria, recruitment scripts, and survey instructions. The same survey link and recruitment procedures were used in both classrooms and cafeteria entrances. We compared key demographic and behavioral variables between the 2 groups and found no significant differences (all *P*>.05), confirming comparable sample composition.

### Translation and Cultural Adaptation of the Measurements

The original English instruments were translated into Chinese following established cross-cultural scale adaptation procedures using a forward- and back-translation procedure to ensure conceptual equivalence. The research team first translated all items into Chinese, and the translation was reviewed by a professor specializing in English-Chinese translation to improve linguistic accuracy and clarity. The translation process focused on prioritizing conceptual equivalence over literal translation to ensure that the Chinese items captured the same underlying constructs as the original instruments. An English-major graduate student, who was blind to the original instruments, then independently back-translated the Chinese version into English. The research team compared the back-translated and original versions to identify semantic discrepancies and revised the Chinese wording through discussion until consensus was reached that the items accurately reflected the intended meanings. Finally, cognitive interviews with 3 undergraduate students were conducted to assess item clarity and cultural appropriateness, and minor wording adjustments were made accordingly.

Brief descriptions and examples were provided in the questionnaire to clarify key concepts (eg, leisure-time PA refers to voluntary PAs performed during free time rather than mandatory school activities), ensuring that participants had a consistent understanding of the items.

### Measurements

#### Social Influence on Physical Activity

The Social Influence on Physical Activity Questionnaire [34] was adapted to measure the social media–based social support for LTPA over the previous 12 months [28]. This adapted scale was used in a previous study among college students in the United States with tested validity and a Cronbach α reliability score of 0.88 [28]. Participants were asked to indicate how often their contacts on this social networking service have demonstrated 3 dimensions of social support, rated on a 5-point scale ranging from 1 (never) to 5 (very often).

*Companionship support* refers to participating in an LTPA with another person; a sample item for companionship support was “made plans with you for doing a leisure-time physical activity together.” The Cronbach α for companionship support is 0.924.

*Informational support* refers to providing positive information regarding LTPA. A sample item for informational support was “informed you about the expected positive effects of a leisure-time physical activity on your health.” The Cronbach α for informational support is 0.905.

*Esteem support* refers to encouragement [34]. A sample was “complimented your good skill in a leisure-time physical activity.” The Cronbach α for esteem support was 0.925.

#### TPB Constructs

##### Attitude

Respondents rated their attitudes using semantic differential scales ranging from 1 to 10. “To me, participating in LTPAs is” boring (1) to interesting (10); unenjoyable (1) to enjoyable (10); unpleasant (1) to pleasant (10); bad (1) to good (10); and undesirable (1) to desirable (10); harmful (1) to beneficial (10); useless (1) to useful (10); weak (1) to strong (10); passive (1) to active (10); and foolish (1) to wise (10). These 10 items were used in previous studies to measure attitudes toward LTPA within the undergraduate student population [35,36]. The Cronbach α was 0.948 in this study.

##### Subjective Norm About LTPA From Contacts on SNSs

Scales developed by Courneya et al [37] were modified to evaluate subjective norms from the contacts on “this SNS,” which refers to the SNS that the participant uses the most. Since students’ social networks enlarge after joining SNSs and befriending people they have never met face-to-face, the subjective norm was modified to address all the contacts on “this SNS.” Participants were asked to respond to the question, “I think that if I were to participate in leisure-time physical activity over the next month, my contacts on this social network site would be…” on 3 semantic differential items: disapproving-approving, unsupportive-supportive, and discouraging-encouraging. Each item was rated on a 5-point scale from 1 to 5. The Cronbach α was 0.925. Higher scores indicate students’ perceptions that their contacts on SNSs would be more approving of their participation in LTPA.

##### Perceived Behavioral Control

The study adopted an existing instrument previously used with the undergraduate student population [35,36]. Participants were asked to indicate the extent to which they agreed with 2 items for LTPA: “I have the resources required to engage in leisure-time physical activity” and “it is easy for me to engage in leisure-time physical activity.” The 5-point Likert-scale ranged from 1 (strongly disagree) to 5 (strongly agree). The Cronbach α was 0.722. A higher score indicated higher perceived behavioral control.

##### Dependent Variable LTPA Intention

Intention was measured by adapting the scale developed by Courneya et al [37]. The reliability for the original 7-point scales ranged from 0.95 to 0.96 [37]. In this study, participants were asked to answer the following questions: (1) How motivated are you to participate in leisure-time physical activity over the next month? (1=not at all motivated, 7=extremely motivated), (2) How strongly do you intend to do everything you can to participate in any leisure-time physical activity over the next month? (1=do not intend, 7=strongly intend), and (3) How committed are you to doing any leisure-time physical activity over the next month? (1=not at all committed, 7=completely committed). The Cronbach α was 0.949. A higher score indicated a higher intention to participate in LTPA.

### Covariates

We also measured several covariates. Gender and grade level were self-reported demographic variables. Average daily time spent on social networking sites was assessed by asking participants to report the approximate number of minutes they spent on SNSs per day. Participants were asked to fill in the blanks of hours and minutes to indicate how long they used the social media platform that they used the most on an average day in total. The hours were then converted to minutes to obtain the total duration.

Weekly participation in LTPA was measured by asking participants to report the total amount of time they usually spent on PAs during their leisure time in a typical week (eg, sports, exercise, or fitness activities outside of school requirements).

The frequency of discussing LTPA on SNS was assessed with the question, “How often have you talked about leisure-time PA on this SNS before the beginning of this semester?” The answers were measured on a 5-point scale ranging from 1 (never) to 5 (very often).

Perceived importance of the contacts on SNS and perceived closeness with them were assessed using single-item ratings asking participants to evaluate (1) “Overall, how important are all your contacts to you?” and (2) “Overall, how close do you feel to all your contacts?”, with each measured on a 7-point Likert scale ranging from 1 (not at all) to 7 (very much).

In addition, height and weight were self-reported and used to compute BMI. Based on the calculated BMI values, participants were categorized into 4 groups: underweight (<18.5), normal weight (18.5-23.9), overweight (24.0-27.9), and obese (≥28.0), and this categorization was also included as a covariate.

### Analysis

Data screening was conducted prior to analysis. Cases with more than 20% missing data were excluded to ensure data quality, and the remaining missing responses were retained as missing values (NA). After screening, 310 valid observations were included in subsequent analyses.

We conducted all analyses in RStudio (R version 4.5.2) [38,39] and used the *lavaan* package (version 0.6‐19) [40,41] for reliability and validity tests and structural equation modeling. Missing data were handled using full information maximum likelihood estimation when applying the *lavaan* package, which allows cases with partially missing responses to be retained and uses all available information to estimate model parameters.

A structural equation model was constructed based on hypotheses about relationships among 7 latent variables: companionship support, self-esteem support, informational support, attitudes, perceived behavioral control, subjective norms, and leisure sports intentions. The model included 3 independent variables (companionship support, informational support, self-esteem support), 3 mediating variables (attitudes, subjective norms, perceived behavioral control), 8 control variables (gender, grade level, daily time spent on SNS, weekly duration of LTPA, frequency of discussing LTPA, perceived importance and closeness of these contacts, BMI), and LTPA intentions as the dependent variable.

Since the control variables were ordinal, the structural equation model was estimated using the diagonally weighted least squares method. The scaled model fit indices showed that *χ*²_720_=1291.5, comparative fit index (0.950), and Tucker-Lewis index (0.944), root mean square error of approximation (RMSEA; 0.047), robust RMSEA (0.047), and standardized root mean square residual (0.063) were all within ideal ranges.

### Ethical Considerations

#### Ethics Approval

The ethical approval for this study was obtained prior to data collection from the San Jose State University IRB on February 9, 2022. The IRB application number is 22027.

#### Informed Consent

The consent notice (written consent document, unsigned by the participant) was on the cover page of our online Qualtrics survey, and students had the opportunity to complete it prior to beginning the survey.

#### Privacy and Confidentiality

All collected data were anonymous and treated as confidential. Questionnaires were administered in secure online formats, and the data were stored in password-protected systems accessible only to authorized researchers.

#### Compensation

Five yuan (RMB, approximately equal to US $0.7 according to the exchange rate of June 5, 2026) was provided to participants for their involvement in this study.

## Results

### Reliability Test of the Measurements

All constructs exhibited satisfactory reliability (Cronbach *α* >0.7). In confirmatory factor analysis, all factor loadings exceeded 0.7, average variance extracted exceeded 0.5, and composite reliability exceeded 0.7, supporting adequate convergent validity, as shown in (Table 1). Correlation coefficients between all factors did not exceed the square root of the factor average variance extracted, indicating good discriminant validity. Model fit indices showed that *χ*²_474_=1194.5, comparative fit index (0.922), root mean square error of approximation (0.070), incremental fit index (0.923), and Tucker-Lewis index (0.913) were all within ideal ranges, while Normed Fit Index (0.878) was slightly below the ideal value. Overall, the model fit was good.

**Table 1.** Confirmatory factor analysis results.

Latent variable	Items	AVE^a^	CR^b^	Cronbach α (95% CI)
Attitudes	10	0.654	0.949	0.948 (0.934-0.960)
Subjective norms	3	0.806	0.926	0.925 (0.892-0.952)
Perceived behavioral control	2	0.588	0.737	0.722 (0.624-0.862)
Companionship support	5	0.709	0.924	0.924 (0.906-0.937)
Informational support	5	0.664	0.907	0.905 (0.879-0.926)
Self-esteem support	5	0.730	0.931	0.925 (0.916-0.946)
LTPA^c^ intentions	3	0.861	0.949	0.949 (0.929-0.965)

a AVE: average variance extracted.

b CR: composite reliability.

c LTPA: leisure-time physical activity.

### Descriptive Analysis Results

A total of 310 valid responses were included in the final analysis, with an effective response rate of 72.1%. The average age of the 310 respondents was 23.03 (SD 3.16) years. A total of 171 (55.2%) participants were male, and 139 (44.8%) participants were female. Social media use was ubiquitous among participants. Most participants (n=271, 87.4%) had more than 2 years of social media experience, and nearly 9 out of 10 (n=278, 89.8%) reported accessing social media multiple times per day. On average, participants spent 126 (SD 131.24) minutes on social media each day and had approximately 267 contacts on their primary platform. WeChat and QQ were identified as the most commonly used platforms (n=268, 86.6%). Around 70% (n=217) indicated that they had engaged in discussing LTPAs on social media in the past week. On average, respondents reported exercising 41.88 (SD 69) minutes moderately and 30 (SD 49) minutes doing vigorous exercise weekly. A total of 309 participants reported their height and weight data, of whom 198 (64.1%) were classified as normal, 56 (18.1%) underweight, 42 (13.6%) overweight, and 13 (4.2%) obese.

### Correlation Analysis Results

Table 2 presents the descriptive statistics and correlations among the study variables. The 3 dimensions of social support, companionship support, informational support, and self-esteem support, were significantly positively intercorrelated (*r*=0.69-0.77; *P*<.001). Companionship support was positively associated with attitudes toward LTPA (*r*=0.26; *P*<.001), subjective norms (*r*=0.15; *P*=.01), perceived behavioral control (*r*=0.13; *P*=.02), and LTPA intentions (*r*=0.42; *P*<.001). Informational support and self-esteem support were also positively correlated with LTPA intentions (*r*=0.22 and 0.38, respectively; *P*<.001).

**Table 2.** Descriptive statistics of variables and correlation matrix.

Variables	Mean	SD	1	2	3	4	5	6	7
Companionship support	2.74	0.95	—						
Informational support	2.71	0.91	0.76^a^	—					
Self-esteem support	2.51	1.00	0.77^a^	0.69^a^	—				
Attitude	7.76	1.74	0.26^a^	0.08	0.24^a^	—			
Subjective norms	7.73	1.85	0.15^b^	0.09	0.12^b^	0.58^a^	—		
Perceived behavioral control	3.57	0.76	0.13^b^	0.04	0.12^b^	0.30^a^	0.26^a^	—	
LTPA^c^ intentions	6.97	2.51	0.42^a^	0.22^a^	0.38^a^	0.61^a^	0.31^a^	0.32^a^	—

a *P*<.001.

b *P*<.05.

c LTPA: leisure-time physical activity.

Consistent with the TPB, attitude was positively associated with subjective norms (*r*=0.58; *P*<.001), perceived behavioral control (*r*=0.30; *P*<.001), and LTPA intentions (*r*=0.61; *P*<.001). Subjective norms (*r*=0.31; *P*<.001) and perceived behavioral control (*r*=0.32; *P*<.001) were also positively related to LTPA intentions. Overall, the correlations among variables were in the expected directions and of moderate magnitude.

### Structural Equation Modeling Results

Overall, as presented in Table 3, the 3 types of social support had a significant positive total effect on LTPA intention (β=.360, 95% CI 0.360-1.244; *P*<.001), although their direct and indirect pathways differed. Companionship support exerted a positive direct effect on LTPA intention (β=.348, 95% CI 0.262-1.511; *P*=.005) and also an additional positive indirect effect through attitude (β=.286, 95% CI 0.274-1.185; *P*=.002). Self-esteem support also showed a positive indirect effect via attitude (β=.138, 95% CI 0.050-0.618; *P*=.03). In contrast, informational support showed a negative indirect effect via attitude (β=−.291, 95% CI –1.154 to –0.372; *P*<.001), revealing a suppression effect that the total positive impact of social support was attenuated by the negative indirect influence of informational support, resulting in a smaller total effect than that of companionship support alone. Meanwhile, self-esteem support had no significant effect on LTPA intention.

**Table 3.** Model path testing results.

Tested path	β (95% CI)	*P* value
Direct effects		
Companionship support→LTPA^a^ intention	.348 (1.511 to 0.262)	.005
Informational support→LTPA intention	−.191 (0.083 to –1.084)	.09
Esteem support→LTPA intention	.118 (0.564 to –0.386)	.17
Indirect effects		
Companionship support→attitude→LTPA intention	.286 (1.185 to 0.274)	.002
Companionship support→subjective norms→LTPA intention	−.022 (0.064 to –0.022)	.36
Companionship support→PBC^b^→LTPA intention	.044 (0.254 to –0.029)	.12
Informational support→attitude→LTPA intention	−.291 (−0.372 to –1.154)	<.001
Informational support→subjective norms→LTPA intention	.008 (0.032 to –0.228)	.59
Informational support→PBC→LTPA intention	−.037 (0.032 to –0.228)	.14
Esteem support→attitude→LTPA intention	.138 (0.618 to 0.050)	.02
Esteem support→subjective norms→exercise intention	−.003 (0.059 to –0.072)	.85
Esteem support→PBC→exercise intention	−.037 (0.032 to –0.228)	.14
Total effect of all types of social support	.360 (1.244 to 0.360)	<.001

a LTPA: leisure-time physical activity.

b PBC: perceived behavioral control.

## Discussion

### Overview

This study contributed to the literature by applying the TPB as a guiding theoretical framework to systematically examine both the direct and indirect effects of various dimensions of social support on social media on LTPA intentions among Chinese college students. We found that different dimensions of social support had various direct and indirect associations with the intention to participate in LTPA among Chinese college students.

### Companionship Support on Social Media

Companionship support demonstrated both significant direct and indirect effects via attitude on LTPA intention, underscoring its strong motivational role in shaping behavioral intentions. Regarding the direct effect, our finding was consistent with previous studies. Previous research has demonstrated that college students’ peer relationships, which are the interpersonal dynamics that emerge when individuals of a similar age or level of psychological development engage in shared activities or collaborative endeavors [42], can significantly and positively predict behavioral intentions to exercise [14,43]. In particular, the findings of Zhou et al [14] suggest a chained mechanism in which peer relationships can become a social support, which then fosters exercise self-efficacy, ultimately influencing intention.

This is one of the first studies to identify that companionship support on social media had an indirect association with LTPA intention via attitude. This is consistent with a previous study that found social support for LTPA on social media has an indirect effect on intention through both affective and instrumental attitude [28].

Unexpectedly, we did not find an indirect effect of companionship support on intention via PBC. This finding is inconsistent with a previous study by Zhou et al [14], where the mediating effect of social support on PA intention via self-efficacy was found. The inconsistency may arise from differences in measurement. Although self-efficacy is a core subcomponent of PBC in the TPB, our study measured general PBC, while Zhou et al [14] focused specifically on exercise self-efficacy. In addition, our study was conducted in a social media context, whereas Zhou et al [14] examined offline peer relationships, which may strengthen self-efficacy more directly. These contextual and measurement differences may explain the distinct findings.

### Informational Support on Social Media

#### Overview

Our study revealed an unexpected pattern in the form of an inverse relationship between informational support and intentions to engage in LTPA, with an indirect effect mediated by attitude. The finding contrasts with previous research that has found that informational support effectively enhances exercise intention primarily by fostering positive attitudes, particularly around its associated benefits. For example, in the context of fitness apps, informational support had a strong indirect association with exercise intentions through the mediation of attitudes toward exercise [30].

The negative indirect effect may be driven by the low credibility of user-generated content on social media. In social media environments, high volumes of user-generated health-related content make it difficult to distinguish misinformation from credible content [44-46]. In the context of COVID-19, during which conflicting or inaccurate information proliferated [47,48], misinformation impeded users’ ability to effectively appraise information or verify its credibility [45,46]. This explanation is also supported by previous literature which noted that adolescents favor peer-sourced information in reliable settings [49]. Hence, Chinese college students may distrust unvetted user-generated exercise content shared by peers on social media due to inadequate source verification and widespread misinformation. Such distrust may reduce positive attitudes toward PA, thereby lowering LTPA intentions.

Possibly, the negative association between informational support and behavioral intentions may also be driven by digital information overload, a phenomenon where available information exceeds individual cognitive processing capacity [50]. Research demonstrates that information overload can lead to heightened levels of fatigue, frustration, and increased information avoidance among social media users [25,50]. Within health contexts specifically, information overload can negatively impact health self-efficacy and increase anxiety levels [25]. Furthermore, information overload further prompts news avoidance and a greater propensity to share health misinformation, ultimately perpetuating an “infodemic” environment that erodes trust in official health messaging [45,46,51]. Thus, the overload of information about PA on social media might lead to Chinese college students’ tendency to avoid the information which might affect their LTPA intention.

#### Self-Esteem Support on Social Media

Our findings indicated a significant indirect effect of self-esteem support on LTPA intentions via attitude. This finding aligns with existing research evidence that self-esteem has a positive effect on attitude and intention toward physical exercise [52,53]. Some previous studies have found that self-esteem support is more strongly associated with actual PA behaviors than with behavioral intentions, whereas other studies have observed significant direct effects of esteem support on exercise intentions, particularly among young women or in the context of mobile health fitness apps [30,54]. Despite the inconsistency of the findings, our study suggested that self-esteem support may function more effectively to reinforce behavioral intention rather than as a requisite antecedent for initiating behavioral change directly.

### Practical Implications

Overall, the results of this study demonstrated how the ubiquity of online social interaction may have altered traditional social support mechanisms. Our findings have several implications for the design of future PA promotion interventions on social media. First, social media interventions aimed at increasing LTPA among Chinese college students should prioritize peer-based companionship features such as group exercise communities, group challenges, and opportunities for interactive feedback rather than the passive dissemination of information. Furthermore, the delivery of informational support on social media for Chinese college students should be sourced from credible and diverse providers (eg, universities, professional sports institutions, certified health experts, or reliable health organizations). To reduce information overload, interventions should provide personalized and actionable content rather than overly comprehensive educational materials. Finally, esteem support strategies may be effectively implemented as postbehavior reinforcement, possibly as part of a phased intervention approach that adapts support types according to various stages of behavioral change.

### Limitations and Future Research Directions

Several limitations of this study should be acknowledged. First, the cross-sectional design limits our ability to infer causal relationships among social support on social media, TPB constructs, and LTPA intentions. Although the proposed model was theoretically grounded in TPB, reverse or reciprocal relationships may also exist. Future studies should use longitudinal or experimental designs to better examine causal mechanisms and temporal dynamics.

Second, the sample was recruited using convenience sampling from a single comprehensive university in East China, which may limit the generalizability of the findings to broader populations of Chinese college students. In addition, participants were recruited both through classroom announcements and at cafeteria entrances, which may have introduced self-selection bias, as students with greater interest in social media or PA may have been more likely to participate. Future research should use multisite randomized or stratified sampling approaches across different regions and university types to improve representativeness.

Third, all variables were measured using self-reported questionnaires, which may be subject to recall bias, social desirability bias, and common method variance. Although validated scales were adopted and anonymity was ensured, future studies could incorporate objective behavioral indicators, digital trace data, or multisource assessments to improve measurement accuracy. Also, some measurements can be further improved. In particular, PBC was assessed using only 2 items and primarily reflected perceived resources and ease of participation rather than the broader construct of exercise self-efficacy. This may partially explain the nonsignificant mediating effect of PBC observed in the study. Future research should adopt more comprehensive and culturally validated measurements of self-efficacy and behavioral control.

Finally, although structural equation modeling enabled simultaneous examination of multiple direct and indirect relationships, the analytical approach remains limited by the assumptions of latent variable modeling and the omission of potentially relevant contextual factors. Variables such as personality traits, mental health status, exercise habits, offline social support, and algorithmic exposure to health-related content on social media were not included in this model and may influence LTPA intentions. Future studies could apply longitudinal structural equation modeling, multilevel modeling, or mixed methods approaches to better capture the complex sociotechnical processes underlying PA intentions in digital environments.

### Conclusions

This study demonstrates the unique functional roles of distinct social support dimensions on social media for LTPA among Chinese college students. Companionship support emerged as the most effective facilitator of LTPA intention, through both an indirect effect via attitudes and a direct effect. Esteem support had a positive indirect effect on LTPA intention through attitude, whereas informational support had a negative indirect effect, which requires careful optimization to avoid unintended adverse effects. Public health interventions aiming to promote LTPA should, therefore, consider emphasizing peer-based or group-oriented social engagement and informational or advisory support from health experts or professionals. Future research, potentially qualitative research from a cultural perspective, could be conducted to examine the negative effects of informational support on Chinese college students’ PA intentions.

## Abbreviations

IRB: institutional review board
LTPA: leisure-time physical activity
PA: physical activity
PBC: perceived behavioral control
RMSEA: root mean square error of approximation
TPB: theory of planned behavior
